# Enhancing therapeutic antibody production through amino acid-induced pH shift in Protein A affinity chromatography

**DOI:** 10.3389/fbioe.2025.1567923

**Published:** 2025-04-04

**Authors:** Senzhu Lin, Yue Wang

**Affiliations:** Downstream Process Development (DSPD), WuXi Biologics, Shanghai, China

**Keywords:** pH shift, low pH unstable protein, Protein A chromatography, amino acid-based elution, downstream purification

## Abstract

Protein aggregation, denaturation, and loss of potency often occur during Protein A chromatography due to the harsh acidic conditions required for antibody elution. This study presents a pH shift-based elution strategy that effectively mitigates these issues by introducing amino acid-based elution buffers to create a milder elution environment and increase the final elution pH. By optimizing the combination of pre-elution and elution buffers, the elution pool pH was increased up to 7.2, significantly enhancing protein stability. Among various elution buffers tested, amino acids with non-polar or polar uncharged side chains—such as leucine, glycine, and serine—exhibited the most effective pH transition, resulting in 0.5–2.9 units pH shifts. Additionally, the use of 50 mM Bis-Tris, pH 7.2 as a pre-elution buffer demonstrated the highest capacity for stabilizing pH shifts. The scalability of this approach was validated using a 10 cm diameter column, where yields remained comparable to small-scale experiments, and elution pool stability was able to be maintained for 72 h at 26°C. These findings establish pH-shifting elution as a scalable, cost-effective method for improving the recovery and stability of low pH-unstable therapeutic antibodies in Protein A chromatography.

## Introduction

The employment of the efficient, rapid, and selective Protein A method in therapeutic antibody purification is widely regarded as a key benefit of this procedure (Hober et al., 2007). Protein A serves not only as a ligand for capturing proteins in affinity capture chromatography but also plays a crucial role in the refining phase, effectively removing aggregates, fragments, host cell proteins (HCPs), and DNA (Rathore and Narnawar, 2022). This dual functionality facilitates a streamlined and robust process for antibody purification. As of now, no other chromatographic technique has matched the comprehensive capabilities of Protein A chromatography (Stange et al., 2021). However, a significant drawback of Protein A capture chromatography is the necessity for harsh acidic conditions during elution. These conditions are required to disrupt strong and specific protein-protein interactions to achieve satisfactory yields. Unfortunately, this acidic elution can lead to antibody aggregation and denaturation, resulting in a subsequent loss of efficacy (Arakawa et al., 2023).

Recent advancements have focused on mitigating the harsh acidic conditions typically required in Protein A chromatography. One such method involves pre-filling the elution collection tank with a neutralization buffer to immediately raise the pH of the elution pool, thereby minimizing aggregate formation. However, determining the precise volume of the pre-filling buffer, based on the estimated elution volume before chromatography, adds complexity and reduces the robustness of the process during large-scale manufacturing due to the critical need for controlled mixing to ensure rapid and uniform homogenization (Shukla et al., 2007; Gagnon et al., 2015; FAS et al., 2024). Additionally, elution buffer systems enhanced with stabilizers such as sorbitol, mannitol, trehalose, sucrose, and polyethylene glycol 4000 (PEG4000), or combinations thereof, have been effective in protecting proteins from acid-induced denaturation at the outset of elution and in reducing aggregate formation (Stange et al., 2021; FAS et al., 2024; Carpenter et al., 1999). However, the high cost of these stabilizers, the difficulties in removing their residuals, and their potential to elevate column pressure or lead to resin fouling significantly restrict the broader adoption. Furthermore, using elution buffers with high salt concentrations has shown promise in increasing the elution pH and reducing aggregation (Arakawa et al., 2004; Scheffel and Hober, 2021), but this may necessitate an additional step to remove the excess salt to prevent adverse effects on subsequent polishing steps. Recently, the introduction of Purolite Praesto™ Jetted A50, a commercially available Protein A resin with modified sequences, has been tailored to facilitate elution at a milder pH of approximately 4.6, thereby preventing aggregate formation (FAS et al., 2024).

In this study, we show a facile elution strategy for Protein A chromatography based on amino acid-induced pH shift to control the pH of the final elution pool effectively, protecting the eluted therapeutic antibodies from acidic environment. The results demonstrate that utilizing the elution buffer containing non-polar or polar uncharged amino acids is crucial for facilitating pH shift in the final elution fraction.

## Materials and methods

### Reagents and equipment

Amino acids, including alanine, arginine, asparagine, glutamic acid, glutamine, histidine, leucine, glycine, methionine, phenylalanine, serine, threonine, tryptophan, and valine, along with benzyl alcohol, citric acid, sodium acetate trihydrate, sodium chloride, sodium hydroxide, sodium phosphate monobasic, tris(hydroxymethyl)aminomethane, and tri-sodium citrate dihydrate were sourced from Merck KGaA (Darmstadt, Germany). Acetic acid, hydrochloric acid, 2-morpholinoethanesulfonic acid monohydrate, its sodium salt, and sodium phosphate dibasic were acquired from Avantor Inc. (Radnor, PA, USA). Bis(2-hydroxyethyl)amino-tris(hydroxymethyl)methane and its hydrochloride counterpart were purchased from MilliporeSigma (Burlington, MA, USA).

The BioCore SEC-300 stainless steel column (7.8 × 300 mm) was obtained from NanoChrom (Suzhou, China). Various Protein A resins such as MabSelect SuRe LX and MabSelect PrismA were procured from Cytiva (Uppsala, Sweden), while AT Protein A Diamond affinity resin came from Bestchrom Biosciences Ltd. (Zhejiang, China). Eshmuno A and Amsphere A3 Protein A resins were provided by MilliporeSigma and JSR Life Sciences (Sunnyvale, CA, United States), respectively. Protein A affinity resin UniMab 50HC was sourced from Suzhou NanoMicro Technology Co. Ltd. (Suzhou, China), and Toyopearl AF-rProtein A HC-650F resin was obtained from Tosoh Bioscience LLC (Tokyo, Japan). Detailed specifications for all Protein A resins used in this study are presented in Table 1. Columns such as the Tricorn 5/150 (0.5 cm I.D.), 6.6/400 (0.66 cm I.D.), and BPG 100/500 (10 cm I.D.) were also obtained from Cytiva. Chromatography was carried out using an AKTA Pure™ 150 system (Cytiva, Uppsala, Sweden), managed via UNICORN™ software (version 7.0, Cytiva). pH and conductivity measurements were conducted using the SevenExcellence™ S470 pH/Conductivity meter (Mettler-Toledo, Columbus, OH, USA). Protein concentrations were determined by spectrophotometry at 280 nm using a NanoDrop 2000 spectrophotometer (Thermo Fisher Scientific Inc., Waltham, MA, USA). An Agilent 1260 High-Performance Liquid Chromatography (HPLC) Infinity II system (Agilent Technologies, Santa Clara, CA, USA) was utilized for size exclusion chromatography-HPLC (SEC-HPLC). Non-reduced Capillary Electrophoresis-Sodium Dodecyl Sulfate (CE-NR) analysis was performed with a LabChip GXII Touch HT instrument (PerkinElmer, Waltham, MA, USA). Third generation generic ELISA kit (Cygnus Technologies, Southport, NC, USA) was used for host cell protein (HCP) analysis.

**TABLE 1:** Resin information.

Resin	Matrix	Ligand^a^	Vendor	Diameter (µm)
MabSelect SuRe LX	Rigid, highly cross-linked agarose	Alkali-stabilized, rec. Protein A	Cytiva	85
MabSelect PrismA	Rigid, highly cross-linked agarose	Alkali-stabilized, rec. Protein A	Cytiva	65
AT Protein A Diamond Plus	Rigid, highly cross-linked agarose	Alkali-stabilized, rec. Protein A	Bestchrom	40∼120
Eshmuno^®^ A	Hydrophilic polyvinyl ether	rec. Protein A	MilliporeSigma	50
Amsphere™ A3	Methacrylic polymer (PMMA)	rec. Protein A	JSR Life Sciences	50
UniMab 50HC	Methacrylic polymer (PMMA)	Alkali-stabilized, rec. Protein A	NanoMicro-technology	50
Toyopearl AF-rProtein A HC-650F	Methacrylic polymer (PMMA)	Alkali-stabilized, rec. Protein A	Tosoh	30∼60

^a^ Although the ligands share the names, the mutation site are distinct.

Proteins utilized in this study, detailed in Table 2, were expressed in CHO cells at WuXi Biologics (Shanghai, China) and clarified by two rounds of centrifugation (1000×g for 10 min; 10,000×g for 30 min) to obtain harvest cell culture fluid (HCCF) as the loading material. Semi-purified protein, stored for later use, was also employed once the HCCF stock was depleted. The semi-purified protein was generated via a Protein A capture step after clarification, followed by immediate depth filtration and titration to match the concentration, pH, and conductivity of HCCF. No significant impact on pH shift was observed when comparing semi-purified protein to HCCF. UV280 peak was observed in the flowthrough phase when loading HCCF, attributed to the presence of cell culture media. In contrast, no UV280 peak was detected with semi-purified protein, as the cell culture media had been removed during the purification process.

**TABLE 2:** Molecule information.

Molecule type	Loading material	Molecular mass (kDa)	pI
mAb A	HCCF and Semi-purified	145	8.25
mAb B	Semi-purified	147	8.52
Fc fusion	HCCF	97	8.90

### Protein A chromatography

**FIGURE 1:**
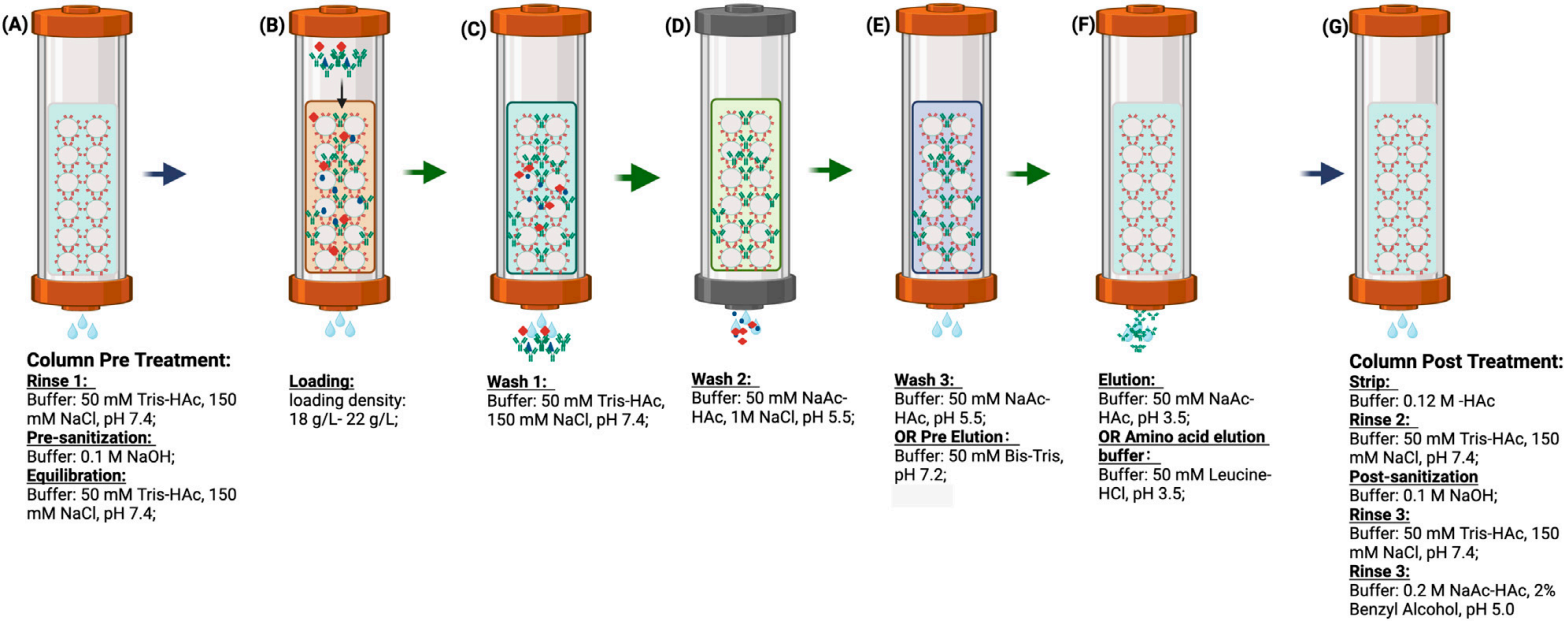
**(A–G)** are the procedure of Protein A chromatography. **(A)** column pre-treatment including rinse 1, pre-sanitization and equilibration; **(B)** protein loading at the column a range 18 g/L to 22 g/L; **(C)** Wash 1 to remove the unbound target protein and impurity; **(D)** Wash 2 for impurity removal; **(E)** Wash 3 for buffer bridging; (F) Elution for target protein recovery; **(G)** column post-treatment including strip, rinse 2, post-sanitization, rinse 3 and storage. In this study, wash 2 was omitted. Wash 3 was redefined as pre-elution and further investigated with different buffer systems. The elution buffer was evaluated using an amino acid buffer system. Additionally, post treatment steps were considered optional in cases where multiple chromatography cycles were performed continuously.

Unless otherwise noted, all Protein A pH shift studies were conducted as described in Figure 1, with a loading density of 20 (18–22) g/L resin. The collection criteria were set above 50 mA/mm. Notably, the traditional impurity removal step—wash 2 in Figure 1 (50 mM NaAc-HAc, 1M NaCl, pH 5.5)—was omitted in most of this study to simplify the procedures but was reintegrated during actual process development to enhance impurity removal. The wash 3 step here (50 mMNaAc-HAc, pH 5.5) was redefined as a pre-elution (50 mM Bis-Tris, pH 7.2), serving dual functions: acting as a bridging buffer to lower salt concentrations and improve elution efficiency, and maintaining the column at a neutral pH for a subsequent pH shift elution. The Protein A load contained HCCF or semi-purified protein. The same volume of wash and pre-elution buffer was applied after loading, with the analysis focusing primarily on the pre-elution and elution phases. After collection, the final elution was assessed based on pH, conductivity, concentration, and volume, along with yield calculation, HCP levels, SEC-HPLC, and CE-NR analysis.

### SEC-HPLC

SEC-HPLC analysis was performed using an Agilent 1260 HPLC system equipped with a BioCore SEC-300 stainless steel column (7.8 × 300 mm). The mobile phase consisted of 50 mM sodium phosphate and 300 mM sodium chloride at pH 6.8. Proteins samples (100 µg) were injected and eluted at a flow rate of 1.0 mL/min, with the eluent monitored by UV absorbance at 280 nm.

### CE-NR

Analytical samples were prepared by mixing the analyte with N-Ethylmaleimide, SDS, and deionized water. Protein samples, along with standards, blanks, and a ladder, were incubated at 70°C for 10 min in a heating block. The processed samples were then analyzed using the LabChip GXII Touch HT instrument, equipped with the High Throughput Protein Express LabChip. For each run, 2 μg of sample was injected.

### HCP measurement

HCP levels in the Protein A eluate were quantified using a third generation generic ELISA kit from Cygnus Technologies (Southport, NC, USA), following the manufacturer’s protocol. Absorbance was measured at 450 nm (primary wavelength) and 650 nm (reference wavelength) using an M5e Microplate Reader (Molecular Devices, San Jose, CA, USA).

## Results and discussion

### Protein A chromatography with pH shifting elution

**FIGURE 2:**
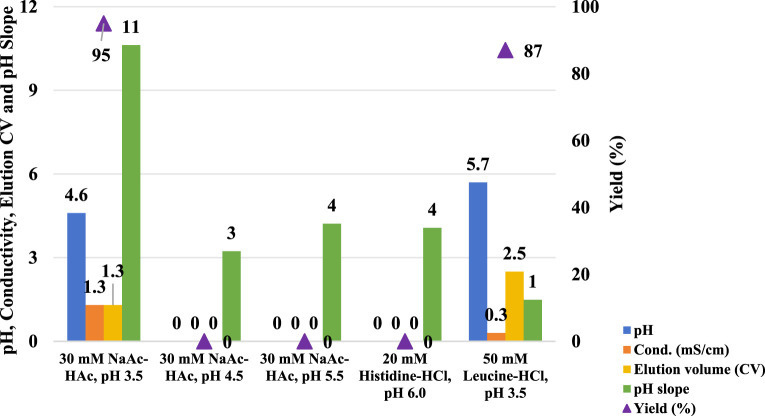
pH shift elution analysis of Protein A. This figure presents the pH, conductivity, elution CV, pH slope, and yield of Protein A chromatography using different elution buffers, including 30 mM NaAc-HAc (pH 3.5, 4.5, and 5.5), 20 mM Histidine-HCl (pH 6.0), and 50 mM Leucine-HCl (pH 3.5). The left Y-axis represents pH, conductivity, elution CV, and pH slope, while the right Y-axis corresponds to yield. The results indicate that elution with 30 mM NaAc-HAc (pH 4.5 and 5.5) and 20 mM Histidine-HCl (pH 6.0) failed to generate measurable values for pH, conductivity, elution CV, and yield, suggesting insufficient elution strength. In contrast, Leucine-HCl (pH 3.5) facilitated a higher elution pH and a lower pH slope, indicating a more gradual and controlled pH transition.

A column with a 0.66 cm diameter and a 26.5 cm bed height was packed with the MabSelect SuRe LX resin before the following five chromatographic runs. HCCF of mAb A was loaded into the column, and experiments were carried out as outlined in Figure 1 with employing five different elution buffers detailed in Figure 2.

**FIGURE 3:**
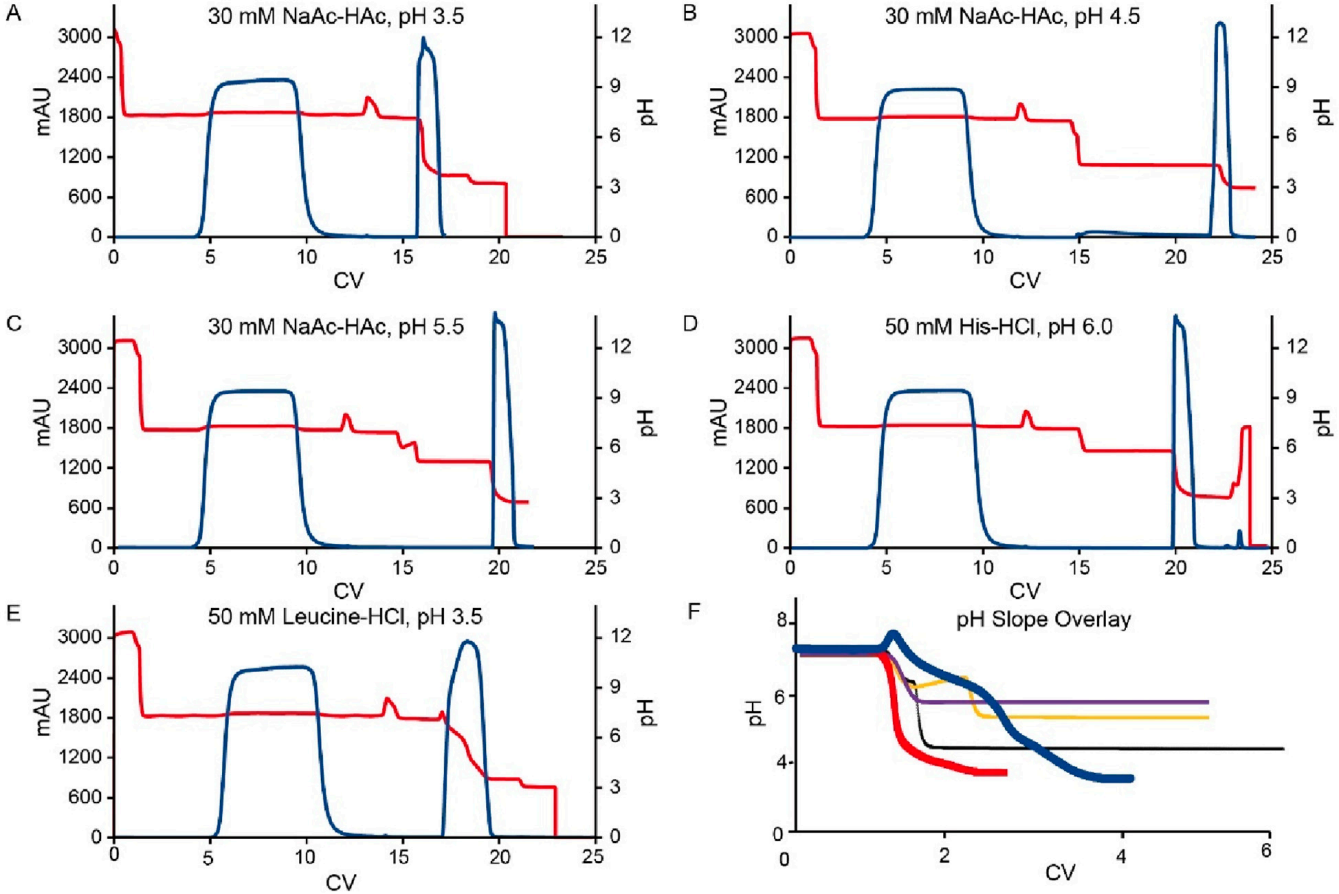
Chromatograms for the Protein A pH shift elution. **(A–E)** are the chromatograms of Protein A pH shift elution with different elution buffer system. The Left Y-axis indicates the UV value and the right Y-axis indicates the pH value. Red line indicates pH profile and blue line indicates UV profile. Elution buffer system includes: **(A)** 30 mM NaAc-HAc, pH 3.5; **(B)** 30 mM NaAc-HAc, pH 4.5; **(C)** 30 mM NaAc-HAc, pH 5.5; **(D)** 20 mM Histidine-HCl, pH 6.0; **(E)** 50 mM Leucine-HCl, pH 3.5; **(F)** is the overlay of elution pH profiles from A to **(E)**. The red line is 30 mM NaAc-HAc, pH 3.5 (slop fit equation: y = −10.623x + 220.37; R^2^ = 0.9538) while the blue line is 50 mM Leucine-HCl indicating an obvious gentler pH slop (slop fit equation: y = −1.4895x + 37.369; R^2^ = 0.9659). The black, yellow and purple lines are 30 mM NaAc-HAc, pH 4.5 (slop fit equation: y = −3.4257x + 75.992; R^2^ = 0.9774), 30 mM NaAc-HAc, pH 5.5 (slop fit equation: y = −4.2187x + 91.941; R^2^ = 0.993) and 20 mM Histidine-HCl, pH 6.0 (slop fit equation: y = −4.0702x + 89.056; R^2^ = 0.9861) respectively.

Compared to the use of a 30 mM sodium acetate-acetic acid (NaAc-HAc) pH 3.5, increasing the pH of the elution buffer to pH 4.5, 5.5, and 6.0 did not enhance the pH of the final elution fraction. Instead, it significantly diminished protein recovery efficiency (Figure 2). Although a UV absorption peak was appeared, it was observed during the strip phase (Figure 3B–D) rather than the elution phase (Figure 3A **)**. Conversely, the amino acid-based elution buffer (50 mM Leucine-HCl, pH 3.5) resulted in an increase in pH values and decent conductivity of the elution fraction, as well as a doubling in the column volume (CV) which indicating a progressive elution (Figure 2). The difference in yield between NaAc-HAc, pH 3.5 and 50 mM Leucine-HCl, pH 3.5 may be due to titer or concentration variations, unexpected breakthrough, or protein entrapment on the resin during room temperature holding. However, these differences were not caused by the gentler pH shift elution, as no UV absorption peaks at 280 nm were detected during post-treatment (Figure 3E). Similar phenomena were observed in subsequent studies, emphasizing the need to use fresh material to minimize variability in future experiments. The comparable yields achieved with the amino acid elution buffer further support its efficacy, as evidenced by a consistent elution profile at 280 nm (Figure 3A, E). The pH decline in the chromatograms for the leucine elution buffer followed a gentler slope compared to the steeper drop observed with NaAc-HAc, pH 3.5 (Figure 3F). Linear trendline analysis confirmed this difference, with the slope value estimated from Figures 2, 3, further validating the milder pH transition with 50 mM Leucine-HCl, pH 3.5. This gradual pH shift likely contributes to a less harsh environment for proteins, leading to an increased final pH and a larger elution volume. Previous findings indicate that amino acid buffers tend to donate H^+^ (Brown and Grimaud, 2023), whereas sodium acetate buffers function as strong base/weak acid systems, retaining H^+^ in the mobile phase during elution. This distinction may explain the observed differences in pH shift profiles and final elution pH. Additionally, the lower conductivity of the elution fraction is likely due to the intrinsically lower conductivity of amino acid buffers compared to sodium acetate buffers. These results showed that amino acid-based elution buffers offer an effective strategy for achieving a progressive elution profile and maintaining a milder pH environment, ultimately enhancing protein stability.

### Effects of resin matrix and ligand

**FIGURE 4:**
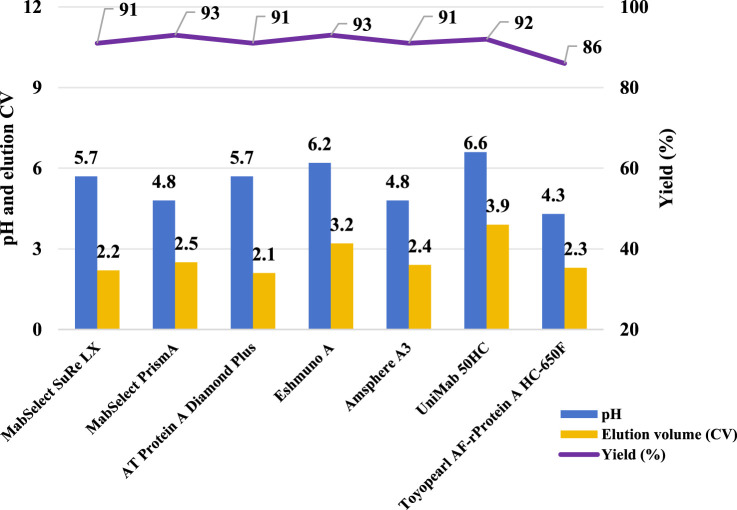
pH shift elution with multiple Protein A resins. pH, elution CV and yield of Protein A chromatography with resin MabSelect SuRe LX, MabSelect PrismA, AT Protein A Diamond Plus, Eshmuno^®^ A, AmsphereTM A3, UniMab 50HC and Toyopearl AF-rProtein A HC-650F. The left Y-axis is for pH and elution CV. Right Y-axis is for yield. Blue column: pH; Yellow column: elution CV(column volume); Purple line: yield.

**FIGURE 5:**
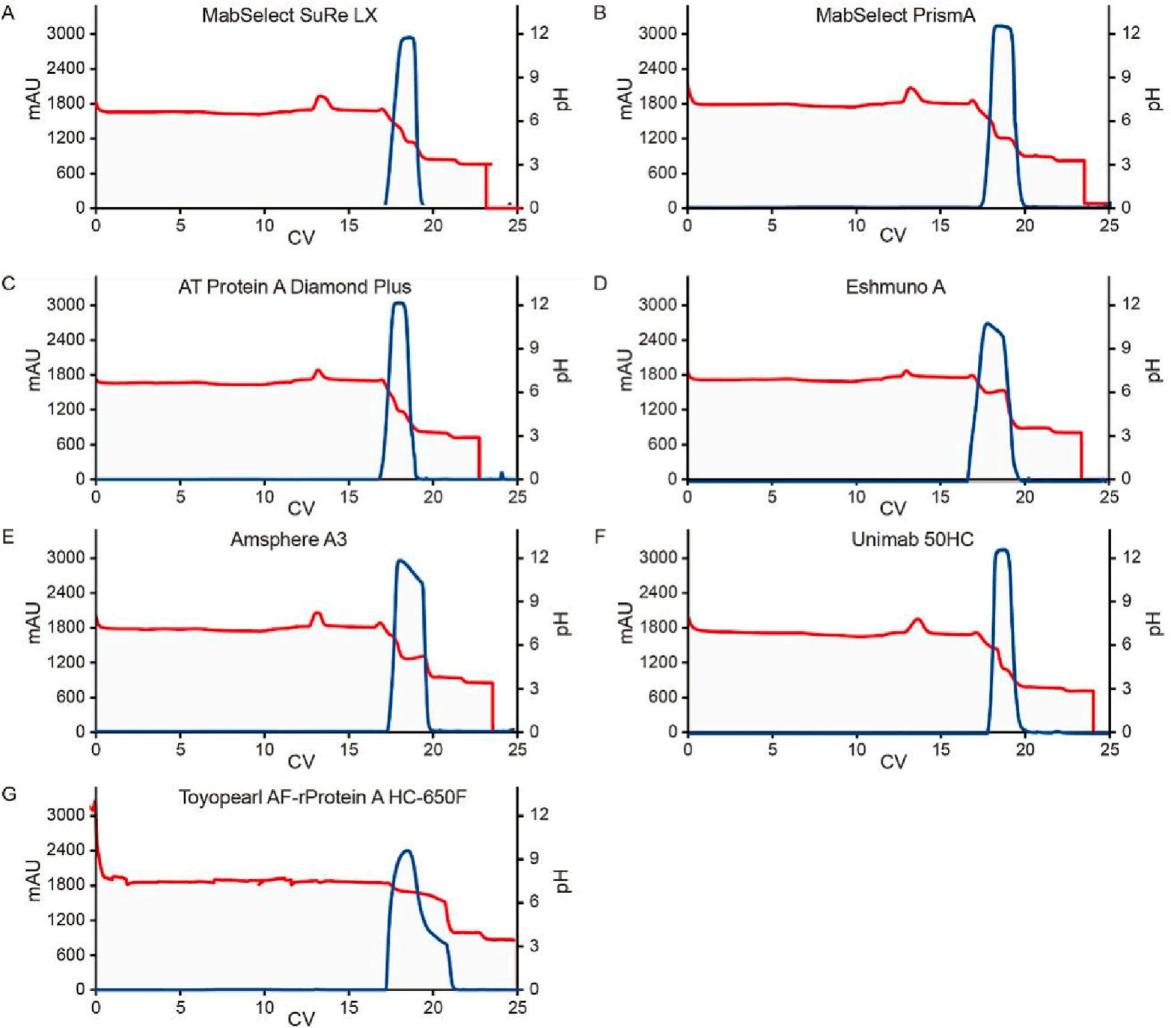
Chromatograms for the pH transition elution with multiple Protein A resins. Chromatograms of Protein A pH shift elution with multiple Protein A resin. Semi purified mAb A was used in the study. The Left Y-axis indicates the UV value and the right Y-axis indicates the pH value. Red line indicates pH profile and blue line indicates UV profile. Reins type includes: **(A)** MabSelect SuRe LX; **(B)** MabSelect PrismA; **(C)** AT Protein A Diamond Plus; **(D)** Eshmuno^®^ A, **(E)** Amsphere™ A3; **(F)** UniMab 50HC; **(G)** Toyopearl AF-rProtein A HC-650F.

In ion exchange chromatography, both functional ligands and charged resin matrices are sensitive to pH transitions, as they interact with H^+^/OH^−^ competition between the stationary and mobile phases (Ghose et al., 2002). To evaluate their impact on pH shift elution, we tested seven different Protein A resins with varying matrices and ligands (Table 1). The resins were packed into 0.5 cm diameter columns (2–3 mL volume), and semi-purified mAb A was used as the model protein. Experiments were conducted following the protocol in Figure 1, using 50 mM leucine-HCl (pH 3.5) as the elution buffer. Interestingly, the final elution pH varied significantly depending on the column matrix (Figure 4). A direct comparison of MabSelect SuRe LX and MabSelect PrismA—both from the same vendor, sharing the same matrix but differing in their ligands (Table 1)—demonstrated that ligand differences significantly influence pH shifting during elution. In contrast, AT Protein A Diamond Plus and MabSelect SuRe LX showed similar pH shifting patterns, suggesting that their Protein A ligands share similar properties. Further evidence of ligand influence was observed in Toyopearl AF-rProtein A HC-650F, Amsphere A3, and UniMab 50HC resins. The final elution pH for Toyopearl AF-rProtein A HC-650F and Amsphere A3 was 4.3 and 4.8, respectively—similar to the standard elution pH obtained with 30 mM NaAc-HAc, pH 3.5. However, UniMab 50HC and Eshmuno A resins produced significantly higher elution pH values of 6.6 and 6.2, respectively. Previous studies suggest that in strong functional ligands, the matrix primarily drives pH transitions, particularly polymethacrylate matrices containing carboxylic groups, whereas crosslinked agarose and polyvinyl ether matrices play a lesser role. Conversely, in the case of weak ion exchangers, the weak functional ligand is the predominant contributor to pH transitions, affecting both polymethacrylate and crosslinked agarose matrices (Ghose et al., 2002). Our results suggest that in this experimental setup, it is the Protein A ligand, rather than the matrix, that acts as a weak functional group on the stationary phase, capturing H^+^ from the mobile phase (amino acid elution buffer) and elevating the pH in the final elution fraction. Despite the gentler elution conditions, yields remained comparable across different resins (Figure 4). Notably, the elution column volume was more consistent in crosslinked agarose matrix resins compared to polymethacrylate and polyvinyl ether matrices. UV absorbance and pH shifting profiles (Figure 5) also varied among resin types but closely aligned with those of the crosslinked agarose matrix resins.

### Impact of target protein

**FIGURE 6:**
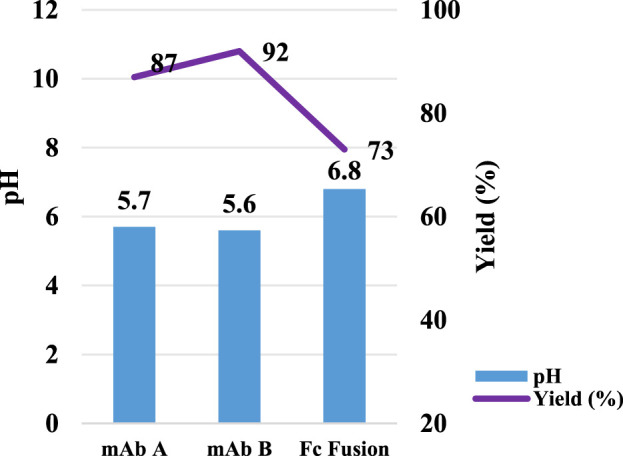
pH transition elution with different molecules. pH and yield of Protein A chromatography with mAb A, mAb B and Fc Fusion. The left Y-axis is for pH. Right Y-axis is for yield. Blue column: pH; Purple line: yield. Lower yield observed in Fc fusion protein may be caused by testing variation since no extra UV peak observed in the chromogram in Figure 7.

**FIGURE 7:**
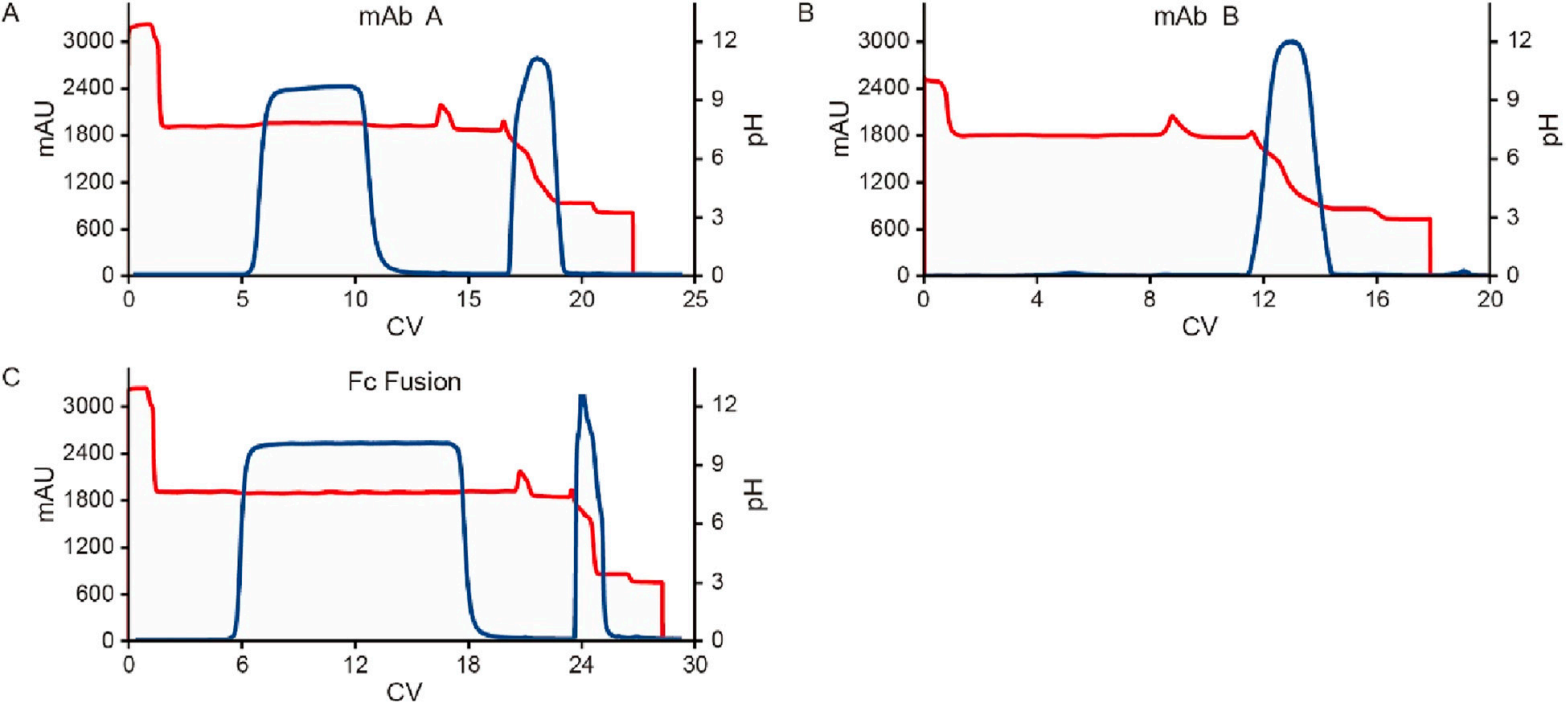
Chromatograms for the pH shift elution with different molecules. Chromatograms of protein A pH shift elution with different molecules. HCCF of mAb A, Fc fusion protein, and semi-purified mAb B were used in the study. The Left Y-axis indicates the UV value and the right Y-axis indicates the pH value. Red line indicates pH profile and blue line indicates UV profile. Molecules includes: **(A)** mAb A; **(B)** mAb B; **(C)** Fc Fusion.

In Protein A chromatography, the ligand, a 42 kDa protein, functions as a weak functional group, influencing pH shifting during elution (Arakawa et al., 2023). We hypothesized that the target protein, once bound to the Protein A ligand, also contributes to H^+^ capture, acting as a weak stationary phase component and further affecting pH transitions. To investigate this hypothesis, three different proteins—HCCF of mAb A, semi-purified mAb B, and an Fc fusion protein (Table 2)—were tested using the same column setup and experimental procedures as in the resin matrix and ligand impact study. The results revealed noticeable differences in pH shift among these proteins (Figure 6). While mAb A and mAb B exhibited similar final elution pH values (5.7 and 5.6, respectively), the Fc fusion protein resulted in a significantly higher pH of 6.8 (Figure 6). Although the precise mechanism behind these variations requires further investigation, the observed differences support the hypothesis that the target protein itself plays a role in H^+^ capture, influencing the final elution pH. Furthermore, the Fc fusion protein displayed a lower yield, which may be due to experimental variability, as no additional UV absorption peaks were detected during post-treatment analysis (Figure 7). These results emphasize the influence of the target protein on chromatography conditions, particularly in modulating pH transitions during elution.

### Effect of the pre-elution buffer

**FIGURE 8:**
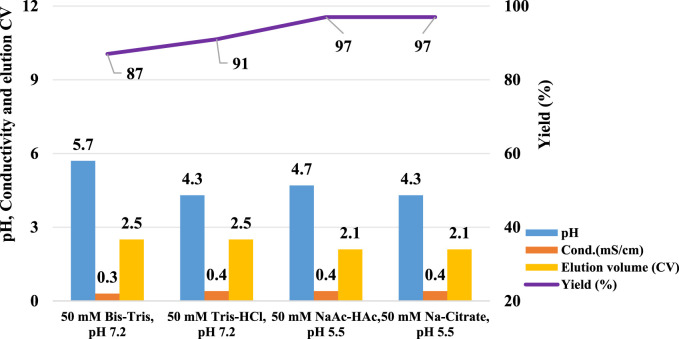
pH transition with different kinds of pre-elution buffer. pH, conductivity, elution CV and yield of Protein A chromatography with pre-elution buffer system 50 mM Bis-Tris, pH 7.2, 50 mM Tris-HCl, pH 7.2, 50 mM NaAc-HAc, pH 5.5 and 50 mM Na-Citrate, pH 5.5. The left Y-axis is for pH, conductivity, and elution CV. Right Y-axis is for yield. Blue column: pH; Orange column: conductivity (mS/cm); Yellow column: elution CV(column volume); Purple line: yield. Blue and green column indicate increasing pH value and lower of pH slop with Leucine-HCl, pH 3.5 as elution buffer.

**FIGURE 9:**
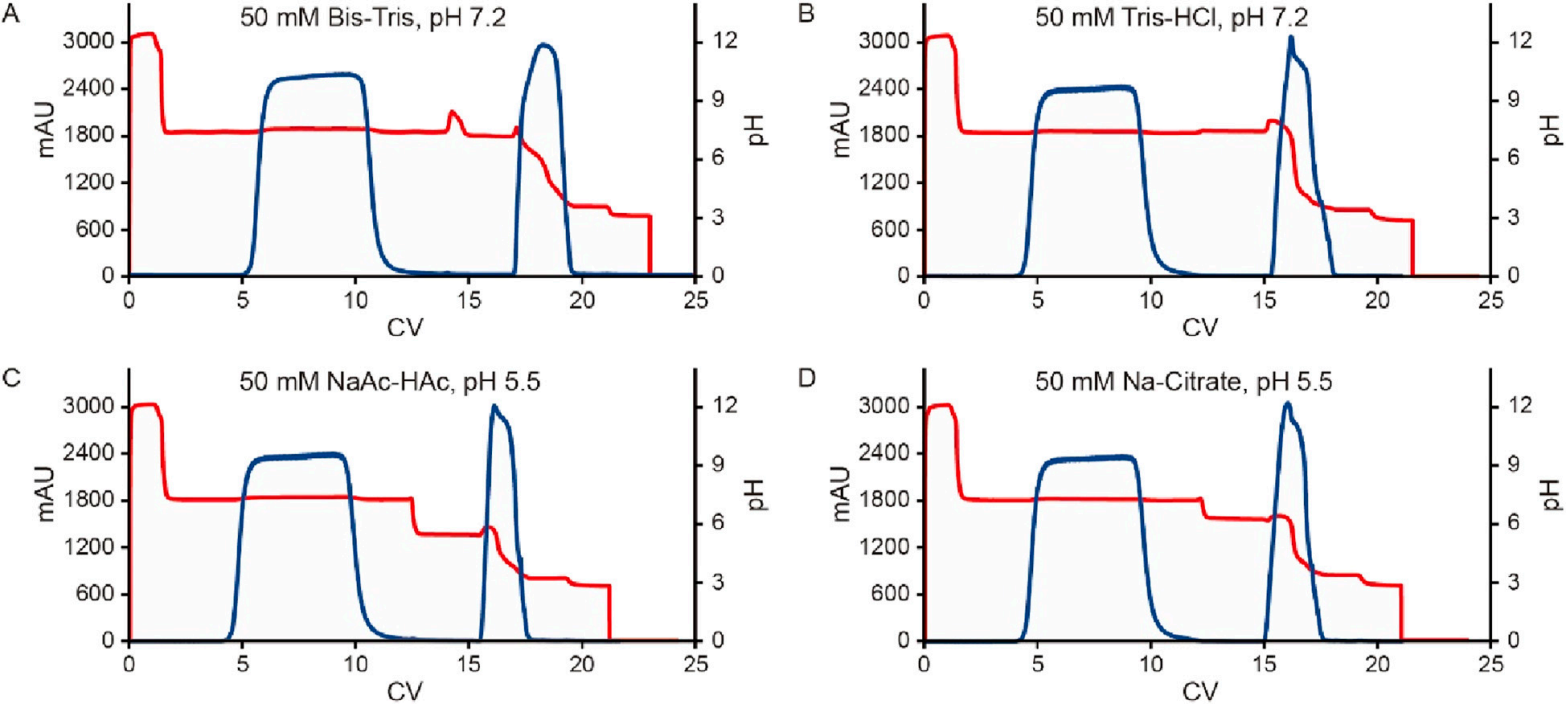
Chromatograms of different kinds of pre-elution buffer. Chromatograms of Protein A pH shift elution with different pre-elution buffers. The Left Y-axis indicates the UV value and the right Y-axis indicates the pH value. Red line indicates pH profile and blue line indicates UV profile. Pre-elution buffer includes: **(A)** 50 mM Bis-Tris, pH 7.2; **(B)** 50 mM Tris-HCl, pH 7.2; **(C)** 50 mM NaAc-HAc, pH 5.5; **(D)** 50 mM Na-Citrate, pH 5.5.

To further investigate the influence of pre-elution buffers on pH transition during elution, we examined three additional commonly used buffers. The clarified harvest fluid of monoclonal antibody A (mAb A) was processed using the standard Protein A chromatography procedure (Figure 1), with modifications to incorporate different pre-elution buffers (Table 3), while maintaining 50 mM leucine-HCl (pH 3.5) as the elution buffer. The results demonstrated comparable elution column volume, yield, pH transitions, and UV profiles across most conditions (Figures 8, 9). However, 50 mM Bis-Tris at pH 7.2 induced a significantly higher pH shift in the elution fraction compared to the other buffers tested. Despite also having a pH of 7.2, 50 mM Tris-HCl showed a lower pH increase, likely due to its buffer capacity being closer to the lower limit of its effective range (pH 7.0–9.0, Table 3). A similar trend was observed with 50 mM Na-Citrate (pH 5.5) and 50 mM NaAc-HAc (pH 5.5). These observations suggest that the stationary phase, once saturated with the pre-elution buffer, undergoes a series of pH-related changes during elution buffer exchange. Initially, the high buffer capacity of the pre-elution buffer stabilizes the pH, resulting in a short plateau at the start of elution. As elution progresses, the stationary phase begins capturing H+ from the mobile phase, triggering a pH increase in the elution fraction, which is reflected in the chromatogram. Eventually, once the stationary phase reaches its H+ capture limit, the elution buffer becomes the dominant factor, leading to a gradual pH decline. These findings highlight the critical role of pre-elution buffer selection in managing pH transitions during Protein A chromatography. Careful consideration of buffer capacity and pH range is essential for optimizing elution conditions and ensuring protein stability.

**TABLE 3:** pH shift pre-elution buffer.

Wash 3	Buffer capacity
50 mM Bis-Tris, pH 7.2	5.8–9.0
50 mM Tris-HCl, pH 7.2	7.0–9.0
50 mM NaAc-HAc, pH 5.5	4.0–6.0
50 mM Na-Citrate, pH 5.5	5.0–7.0

### Effect of amino acids in the elution buffer

**FIGURE 10:**
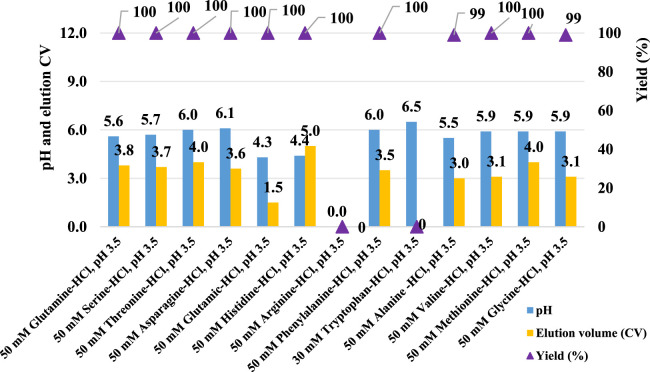
Amino acid buffer for pH shift elution. pH, elution CV and yield of Protein A chromatography with 13 elution buffer systems detailed in Table 4. The left Y-axis is for pH and elution CV. Right Y-axis is for yield. Blue column: pH; Yellow column: elution CV (column volume); Purple triangle: yield. pH, elution CV and yield are zero for elution with buffer 50 mM arginine-HCl, pH 3.5 due to no UV peak shown in elution phase. Elution CV and yield are zero for elution with buffer 30 mM tryptophan-HCl, pH 3.5 due to the abnormal UV platform during elution.

**FIGURE 11:**
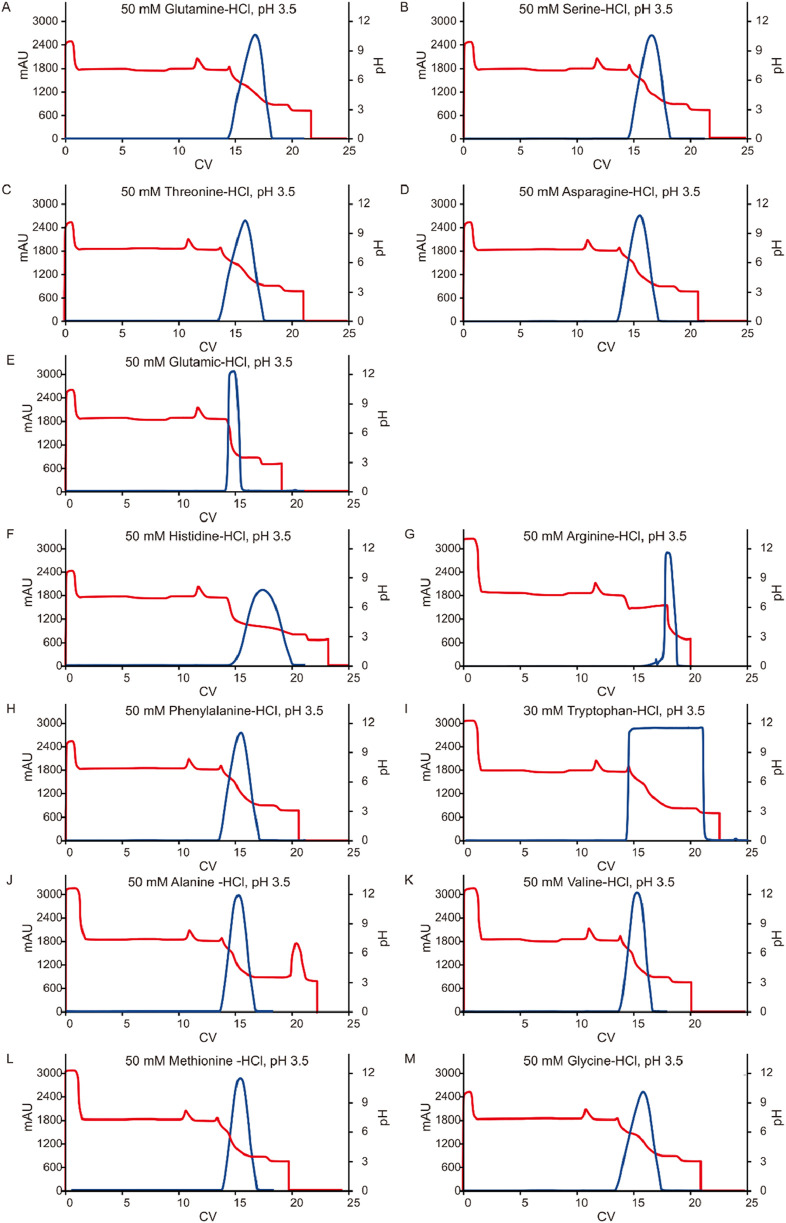
Chromatograms of the pH shift elution with amino acid buffer screening. Chromatograms of Protein A pH shift elution with different elution buffers. While semi purified mAb A was used as model protein. The Left Y-axis indicates the UV value and the right Y-axis indicates the pH value. Red line indicates pH profile and blue line indicates UV profile. Elution buffer includes: **(A)** 50 mM glutamine-HCl, pH 3.5; **(B)** 50 mM serine-HCl, pH 3.5; **(C)** 50 mM threonine-HCl, pH 3.5; **(D)** 50 mM asparagine-HCl, pH 3.5; **(E)** 50 mM glutamic-HCl, pH 3.5; **(F)** 50 mM histidine-HCl, pH 3.5; **(G)** 50 mM arginine-HCl, pH 3.5; **(H)** 50 mM phenylalanine-HCl, pH 3.5; **(I)** 30 mM tryptophan-HCl, pH 3.5; **(J)** 50 mM alanine -HCl, pH 3.5; **(K)** 50 mM valine-HCl, pH 3.5; **(L)** 50 mM methionine-HCl, pH 3.5; **(M)** 50 mM glycine-HCl, pH 3.5.With 30 mM tryptophan-HCl, pH 3.5 as elution buffer, The UV absorption of tryptophan lead to an the abnormal UV absorption during elution and then impacted the elution CV calculation and concentration measurement. With 50 mM arginine-HCl, pH 3.5 as elution buffer, no UV peak appeared in the elution phase but in the strip phase.

Given the significant impact observed from different types of pre-elution buffers, this study also evaluated the effect of various amino acids on pH shifting elution. Thirteen representative amino acids with differing side chain groups, as listed in Table 4, were tested to assess their influence on the pH shift behavior. Semi-purified mAb A was used as the model protein, following the standard procedure outlined in Figure 1. Interestingly, most amino acid-based elution buffers—with the exception of those containing glutamic acid, histidine, and arginine—exhibited comparable pH shifting effects, yield, and elution column volume (Figure 10). This suggests that amino acids with polar uncharged and non-polar side chains (Table 4) are generally well-suited for pH shift elution in Protein A chromatography. However, glutamic acid did not contribute to pH transition, likely because its negatively charged side chain in the mobile phase resists donating H^+^ to the stationary phase, resulting in a lower-than-expected pH shift in the final elution fraction. Conversely, the positively charged side chain of arginine and histidine tended to donate H^+^, maintaining a higher acidity throughout the elution, as evidenced in the chromatogram (Figure 11F–G). Notably, buffers containing amino acids with polar uncharged and non-polar side chains exhibited a pH profile similar to that of the leucine buffer, where pH transitions occurred with a gentle slope. Meanwhile, buffer containing negatively charged amino acids behaved similarly sodium acetate buffer, whereas those with positively charged amino acids (e.g., arginine and histidine) maintained a more acidic pH throughout the elution process. Collectively, the selection of an effective pre-elution buffer is critical for achieving optimal pH transitions, with Bis-Tri proving be the most effective among those tested. Similarly, the choice of amino acid in the elution buffer plays a key role in maintaining flexibility and stability in pH shift elution. Most amino acids with polar uncharged and non-polar side chains induced a 0.5–2.9 units pH shift in the elution fraction, making them suitable candidates for large-scale applications.

**TABLE 4:** Amino acid buffer screening for pH shift elution.

Elution buffer	Amino acid R group property
50 mM glutamine-HCl, pH 3.5	Polar uncharged side chains
50 mM serine-HCl, pH 3.5	
50 mM threonine-HCl, pH 3.5	
50 mM asparagine-HCl, pH 3.5	
50 mM glutamic-HCl, pH 3.5	Negatively charged
50 mM histidine-HCl, pH 3.5	Positively charged
50 mM arginine-HCl, pH 3.5	
50 mM phenylalanine-HCl, pH 3.5	Non-polar side chains
30 mM tryptophan-HCl, pH 3.5 a	
50 mM alanine-HCl, pH 3.5	
50 mM valine-HCl, pH 3.5	
50 mM methionine-HCl, pH 3.5	
50 mM glycine-HCl, pH 3.5	

^a^ The UV, absorption of tryptophan impacts the elution CV, calculation and concentration measurement.

### Effect of column size and the behavior of unstable proteins

**FIGURE 12:**
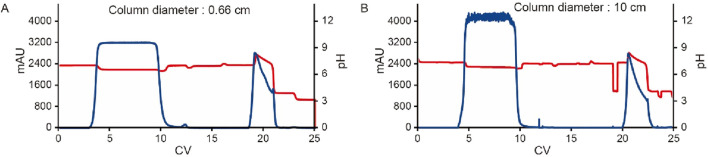
Chromatograms for the pH transition elution scalability study. Chromatograms of Protein A pH shift elution with different column size. Same procedure was followed, the extra CV before elution of the 10 cm column was caused by the additional system wash during large scale. The Left Y-axis indicates the UV value and the right Y-axis indicates the pH value. Red line indicates pH profile and blue line indicates UV profile. Column size includes: **(A)** 0.66 cm, **(B)** 10 cm.

To evaluate the impact of column size on the scale-up performance and the behavior of unstable proteins, the MabSelect SuRe LX resin was packed into columns with diameters of 0.66 cm and 10 cm, both with a bed height of 20 cm. The clarified harvest fluid of an unstable mAb was loaded onto these columns following the same pH shift procedure in Figure 1 with an additional wash 2 step (50 mM Tris, 1 M NaCl pH 7.2) included. The results showed that elution yield, elution volume, chromatogram quality, elution pool pH, SEC, CE-SDS-NR, and HCP levels remained consistent across both column scales (Table 5 and Figure 12). These results suggest that the column size does not significantly affect the pH shift elution strategy, confirming its scalability and robustness. During Protein A chromatography, HCPs exhibited different binding behaviors, affecting their retention and removal efficiency. Nonspecifically attached or weakly bound HCPs were typically removed during the wash 2 step (Figure 1), a process that remains unaffected by the introduction of a pH shift elution strategy. HCPs with weaker or comparable Protein A binding affinity tended to co-elute with the target protein, while those with strong binding affinity require harsher elution conditions to break their interaction with the resin. The introduction of gentler pH elution conditions may cause some of the co-eluting HCPs to shift toward stronger retention, preventing their co-elution with the target protein and thus reducing HCP contamination in the elution fraction. A comparison of low pH elution and pH shift elution strategies supports this hypothesis, as the HCP level measured in the pH shift elution fraction was 446 ppm, compared to 500 ppm in the low pH elution fraction, indicating a modest improvement in HCP removal efficiency (Table 6). To further assess the benefits of the pH transition shift strategy, the stability of the elution fraction from the 10 cm column was examined. For comparison, samples purified using the traditional low pH elution strategy (elution buffer 50 mM NaAc-HAc, pH 3.5 with elution fraction pH at pH 4.3) was also evaluated. The results demonstrated that with the pH shift strategy (elution buffer: 50 mM glycine-HCl, pH 3.5, elution fraction pH 7.2), the high molecular weight (HMW) species remained stable for 72 h at 26°C. In contrast, samples processed using the traditional low pH elution strategy exhibited a 7% increase in HMW species within just 1 hour (Table 6). These results indicate that the pH shift elution strategy is highly effective for capturing low pH-unstable proteins in Protein A chromatography, offering improved protein stability and potential advantages for large-scale manufacturing.

**TABLE 5:** Scalability study of the pH transition elution strategy.

Column size (Diameter(cm)*Heigh(cm))	Elution pool pH	Yield (%)	Elution volume (CV)	SEC_HPLC (Main/HMW/LMW) %	CE-NR (%)	HCP (ppm)
0.66 *20	7.1	88	2.4	88.4/9.5/2.1	96.9	440
10*20	7.2	87	2.4	88.5/9.2/2.3	96.1	446

**TABLE 6:** Stability of pH shift elution fraction of low pH unstable protein.

Holding condition	SEC_HPLC (Main/HMW/LMW) %	
	pH shift elution at pH 3.5 with an elution fraction pH of 7.2^a^	Low pH elution at pH 3.5 with an elution fraction pH of 4.3^a^
26°C, 0 h	88.5/8.9/2.5	88.1/9.0/2.9
26°C, 1 h	NA	81.4/15.8/2.8
26°C, 24 h	88.8/8.7/2.5	NA
26°C, 48 h	88.7/8.7/2.7	NA
26°C, 72 h	88.7/8.7/2.6	NA

^a^ The HCP, is 446 ppm for pH shift elution strategy and 500 ppm for low pH elution strategy.

## Conclusion

This study developed and evaluated a pH shift elution strategy as an effective method for purifying low pH-unstable proteins using Protein A chromatography. The results highlight the critical role of elution and pre-elution buffers in pH transition performance, influenced further by stationary phase properties, including resin type and target protein characteristics. Among the pre-elution buffers tested, the Bis-Tris buffer displayed a substantial advantage by significantly enhancing the pH level in the elution fraction. This suggests that the effectiveness of pH shift elution is likely dependent on the pH level and its position within the buffer capacity range. A higher pH level and a position farther from the buffer range boundary tend to provide greater tolerance and facilitate more effective pH shift. Regarding the selection of elution buffer, amino acids with non-polar or polar uncharged side chains were found to induce a 0.5–2.9 units pH increase in the final elution fraction, making them preferable to buffers containing negatively or positively charged amino acids. The impact of different Protein A resins was also explored to understand how variations in resin matrices or ligands affect pH transitions. Although all tested resins exhibited pH shifts, the influence of the resin matrix was less pronounced compared to that of the ligand. This was further substantiated by experiments with different proteins using the same resin, which demonstrated varied pH transitions even when the matrix and ligand remained constant. Furthermore, the strategy’s scalability and efficacy were validated using a 10 cm diameter column packed with an unstable mAb, underscoring its potential benefits for processing low pH unstable proteins. However, gentler elution conditions, which result in increased elution column volumes and lower elution fraction concentrations, could introduce manufacturing challenges, particularly in container handling, low pH virus inactivation, intermediate depth filtration (int.DF), and anion exchange chromatography (AEX). These challenges can be mitigated by reducing collection volume via UV cutoff at the peak tail, or by integrating tangential flow filtration after Protein A capture to concentrate the elution fraction. For virus inactivation, alternative methods such as solvent/detergent (S/D) treatment at protein-stable pH or low pH with stabilizers can be employed (Stange et al., 2021). Additionally, the low conductivity of amino acid-based elution buffers may improve impurity removal (e.g., HCPs and aggregates) during int.DF and AEX, though potential yield loss should be carefully managed by adjusting NaCl levels to balance yield and impurity clearance efficiency.

Overall, this scalable pH shift strategy, particularly when using Bis-Tris as a pre-elution buffer and amino acids in the elution buffer, offers a viable approach for mitigating harsh low pH elution conditions, thereby enhancing protein stability and yield. Further research should focus on optimizing amino acid concentrations and combinations to maximize the pH-shifting effect while minimizing costs and process complexity. The integration of new buffer systems and automation technologies could improve precision and reproducibility, while extending the strategy to proteins with extreme pH sensitivities could broaden its applicability in biopharmaceutical manufacturing. Additionally, the development of predictive models to simulate and optimize chromatographic interactions under different conditions may provide valuable insights for further refining this approach.

## Data Availability

The raw data supporting the conclusions of this article will be made available by the authors, without undue reservation.
